# Multi-metal contamination shapes abundance, co-occurrence, and mobility potential of resistance and virulence genes in mining-impacted soils

**DOI:** 10.1016/j.imj.2026.100260

**Published:** 2026-05-04

**Authors:** Qitao Zhang, Shixiong Li, Xiaobing Wang, Yuechen Sun, Jingpeng Liu, Jiefei Gao, Chengcheng Deng, Wenlong Zhao, Yixin Ma, Jinrou Quan, Qi Yin, Danyan Jian, Ruihai Zhang, Rui Qi

**Affiliations:** a School of Public Health, Lanzhou University, Lanzhou 730000, China; b Jiayuguan Centers for Diseases Control and Prevention, Jiayuguan 735100, China; c Jiuquan Second People's Hospital, Jiuquan 735000, China

**Keywords:** Metal, Metal resistance gene, Antibiotic resistance gene, Virulence factor gene, Mobility potential

## Abstract

**Background:**

Antimicrobial resistance is a growing global public health concern, posing a serious threat to human health. This study aimed to characterize the composition and distribution of microbial communities, metal resistance genes (MRGs), antibiotic resistance genes (ARGs), and virulence factor genes (VFGs) under multi-metal stress and assess the impacts of metal and soil properties on the diversity, abundance, carrying rate (proportion of gene carriers), co-occurrence rate (proportion of microorganisms co-carrying multiple gene types), and mobility potential (MP, likelihood of horizontal gene transfer) of these genes.

**Methods:**

Soil samples were collected from eight sampling sites within a metal mining area (metal-contaminated soil group, MS) and four sites located more than 3 km away from the mining area (control group). Metal concentrations and physicochemical properties of the soils were measured using standard methods. Metagenomic sequencing was performed to characterize the composition and distribution of the microbiome, resistome, and virulome. Statistical modeling was applied to examine the effects of heavy metal content and soil properties on the relative abundance, co-occurrence, and mobilome potential of the three gene types.

**Results:**

Fe, V, Cr, and Cu primarily promoted the diversity, carrying rate, and co-occurrence rate of microbial communities, MRGs, ARGs, and VFGs. In contrast, Ni and Zn exhibited overall inhibitory effects. For every unit increase in Fe and V, the MP of MRGs and VFGs was associated with an increase of 3.0 × 10⁻⁵ and 1.2 × 10⁻⁵, respectively. A per 1 mg/kg increase in Cr and Cu was correlated with a decrease of 4.3 × 10⁻⁵ and 1.1 × 10⁻⁴ in the MP of ARGs and of MRGs, respectively. Positive correlations were found between the MP of plasmid‑mediated ARGs and Cr, and between transposon‑mediated ARGs and Cr/V. The MP of transposon‑mediated MRGs correlated positively with Fe, while Cu correlated negatively with plasmid‑mediated ARGs but positively with insertion sequence‑mediated ARGs. Ni concentration was positively associated with the MP of IS‑mediated VFGs.

**Conclusions:**

Metals alter the composition and distribution of microbial communities, MRGs, ARGs, and VFGs. A key mechanism underlying this regulation is the modulation of their mobile potential, which either facilitates or restricts horizontal gene transfer.

## Highlights

• High-level metal stress significantly altered microbial community structure and antibiotic resistance gene composition, and decreased metal resistance gene and antibiotic resistance gene diversity.
• Fe, V, Cr, and Cu primarily promoted the relative abundance, richness, carrying rate, and co-occurrence rate of metal resistance genes, antibiotic resistance genes, and virulence factor genes. In contrast, Ni and Zn exhibited overall inhibitory effects.
• Fe, V, and Ni enhanced the overall mobility potential of metal resistance genes, antibiotic resistance genes, and virulence factor genes, respectively.
• Fe and V specifically increased the mobility potential of transposon-mediated metal resistance genes and antibiotic resistance genes, respectively.

## Graphical abstract

**Figure:**
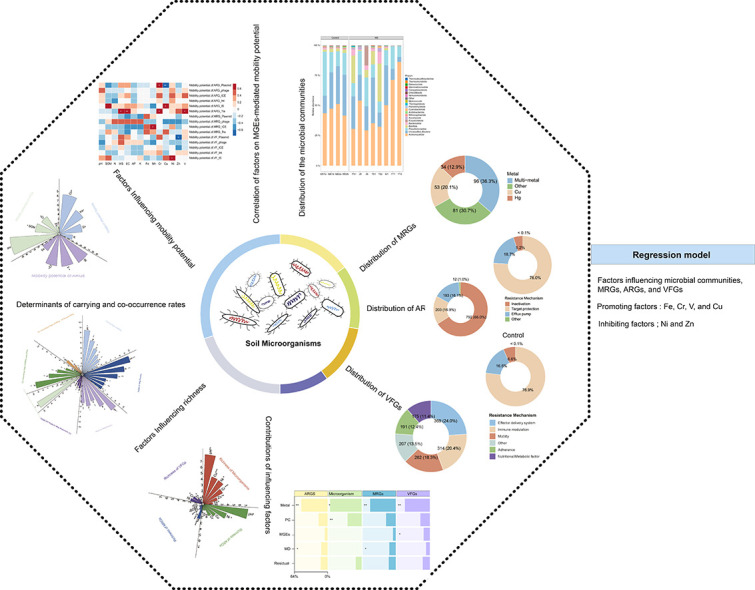

## 1 Introduction

Soil constitutes one of the planet’s largest reservoirs of microorganisms and resistance genes on Earth. In addition to harboring a vast diversity of intrinsic resistance genes, it continuously receives resistant bacteria and resistance genes from human and animal waste.^1^ This role establishes it as a potential pathway for the spread of resistance genes into human environments, posing a severe threat to global public health.^2^, ^3^, ^4^ Heavy metal pollution, resulting from industrial emissions, agricultural activities, and mineral resource extraction, has emerged as a global environmental issue. Heavy metals can significantly promote the development and dissemination of antibiotic resistance through environmental stress and co-selection mechanisms.^5^

Antimicrobial resistance has been identified by the United Nations Environment Programme as one of the six key emerging environmental challenges globally.^6^ It is estimated that infections by drug-resistant bacteria could cause up to 10 million deaths annually by 2050.^7^^,^^8^ Heavy metal exposure promotes the enrichment and dissemination of metal resistance genes (MRGs), antibiotic resistance genes (ARGs), and mobile genetic elements (MGEs).^9^, ^10^, ^11^, ^12^ Microorganisms carrying MRGs gain an ecological advantage under metal stress through efficient detoxification mechanisms. This advantage further facilitates the dissemination of ARGs via co-selection and cross-resistance.^13^ Virulence factor genes (VFGs) encode products that significantly enhance microbial colonization and pathogenicity. Of greater concern is that VFGs, together with MRGs and ARGs, can undergo horizontal gene transfer via MGEs. This process collectively drives the emergence and dissemination of multidrug-resistant pathogens, thereby elevating environmental and public health risks.^14^, ^15^, ^16^, ^17^, ^18^

Previous studies have explored the relationship between heavy metals and antibiotic resistance. For instance, research simulating pig manure application to investigate the effects of copper (Cu) and zinc (Zn) on ARGs and MRGs demonstrated that both metals promoted the proliferation of ARGs in soil but exerted differential effects on MRGs: Cu suppressed their increase, whereas Zn stimulated it.^19^ Furthermore, trace metal ions (e.g., Cu, lead [Pb]) have been shown to enhance the conjugative transfer of ARGs mediated by the RP4 plasmid, while cadmium (Cd) exhibited an inhibitory effect.^20^^,^^21^ However, current research remains largely confined to single-metal exposure or laboratory-scale microcosms, which limits the ability to accurately reflect the interactive mechanisms and ecological effects among the microbiome, resistome, and VFGs under realistic conditions of long-term, multi-metal co-contamination in natural environments.

This study utilized soils from a multi-metal polluted mining area as a natural experimental model. By employing metagenomic sequencing and statistical models in an integrated way, this study was designed to systematically investigate the composition and distribution of the microbial community, MRGs, ARGs, and VFGs under multi-metal stress and the effects of metal and physicochemical properties on the relative abundance, co-occurrence, and mobility potential of these three gene types. This study, conducted at a field scale, analyzed the profiles of ARGs, MRGs and VFGs, defined and assessed the carrying rate (CR), co-occurrence rate (CoR), and mobility potential (MP).

## 2 Materials and methods

### 2.1 Sample collection and processing

Soil samples were collected from the Mazong mountain area in Jiuquan City, Gansu Province. Within the metal mining area, eight sampling sites were designated as the metal-contaminated soil group (MS). To ensure the control group and MS group had similar conditions (light, humidity, etc.) but lower metal concentrations, four sampling sites located more than 3 km away from the mining area were selected as the control group.^22^ At each sampling site, three topsoil subsamples without vegetation cover were collected and thoroughly mixed to form a representative sample. Approximately 500 g of soil was collected from a depth of 0–15 cm using a shovel. Each sample was then stored in a pre-labelled and sealed sampling bag with its information recorded. Subsequently, each sample was divided: One portion was reserved for DNA extraction, while the other portion was air-dried for the determination of physicochemical properties and metal concentrations. Soil samples were analyzed for metal concentrations (iron [Fe], chromium [Cr], vanadium [V], manganese [Mn], Zn, Cu, and nickel [Ni]) and physicochemical properties, including pH, electrical conductivity (EC), total water-soluble salts (WS), soil organic matter (SOM), hydrolytic nitrogen (N), available phosphorus (AP), and available potassium (AK), with the corresponding measurement methods provided in Table S1. The major soil metal Fe is quantified in g/kg, while other trace metals are quantified in mg/kg.

### 2.2 DNA extraction, metagenomic sequencing, and annotation

DNA was extracted from soil samples using the cetyltrimethylammonium bromide method.^23^ Sequencing libraries were constructed using the NEB Next® Ultra™ DNA Library Prep Kit (NEB, USA) for Illumina. Following quality control, qualified DNA was randomly sheared into fragments of approximately 350 bp using a Covaris ultrasonicator (Covaris S2 System, Massachusetts, USA). The insert size of the libraries was assessed using an Agilent 2100 Bioanalyzer (Agilent Technologies Co.Ltd., USA), and concentrations of the libraries were accurately quantified by real-time PCR (Bio-Rad, USA). Index-coded samples were clustered on the cBot Cluster Generation System using the Illumina PE Cluster Kit (Illumina, USA). After cluster generation, the DNA libraries were sequenced on an Illumina Novaseq 6000 platform (Illumina, San Diego, CA, USA), yielding the raw metagenomic data. Raw sequencing data were processed using Trimmomatic to remove low-quality sequences and Bowtie2 to filter out host-derived (human) DNA.^24^^,^^25^ Taxonomic profiling of the clean reads was performed using Kraken 2 against a custom microbial nucleotide database (up to September 10, 2022).^26^ The resulting taxonomic counts were then refined using Bracken to estimate the relative abundance of each organism.^27^ Clean reads were aligned to the BacMet (v2.0), CARD (v4.0.0), and VFDB (v6.0) databases using DIAMOND to determine the composition and relative abundance of MRGs, ARGs, and VFGs in each sample (*E*-value ≤ 1 × 10⁻⁵, identity ≥ 80%).^28^, ^29^, ^30^, ^31^ For the annotation of integrons, transposons, and insertion sequences (IS), a custom database was built from relevant protein sequences extracted from the NCBI NR database (up to April 24, 2025, https://www.ncbi.nlm.nih.gov/protein/), against which clean reads were aligned. Integrative and conjugative elements were annotated using the ICEberg 3.0 database.^32^

### 2.3 Metagenomic assembly and annotation

To obtain contigs containing more complete biological information for inferring gene carriage at the organismal level, clean reads were assembled de novo using MEGAHIT with default parameters.^33^ The assembly quality was assessed with QUAST.^34^ Plasmid sequences were identified using PlasFlow.^35^ Subsequently, contigs ≥ 5000 bp were annotated for phages using VirSorter2.^36^ Open reading frames were predicted from the assembled contigs using Prodigal.^37^ Protein sequences were functionally annotated by alignment against the BacMet, CARD, VFDB, ICEberg 3.0, and a custom MGEs database using DIAMOND.

### 2.4 Definition and calculation of CR, CoR, and MP

This study defined three key metrics: the CR, the CoR, and the MP.

CR is defined as the proportion of microorganisms carrying a specific target gene. It was calculated using the Equation (1):

(1) $$\begin{array}{c} CR_{i}=N_{i}\text{contigs}\left(\text{MRGs}/\text{ARGs}/\text{VFGs}\right)/N_{i}\text{contigs} \end{array}$$

*Nᵢ* contigs (MRGs/ARGs/VFGs) is the number of contigs carrying at least one MRG, ARG or VFG. *Nᵢ* contigs is the total number of contigs.

CoR represents the proportion of microorganisms carrying two or more types of genes simultaneously. The calculation formula is Equation (2):

(2) $$\begin{array}{c} \text{Co}R_{i}=N_{i}\text{contigs}\left(\text{co-occurrence}\right)/N_{i}\text{contigs} \end{array}$$

Where *Nᵢ* contigs (co-occurrence) is the number of contigs carrying at least two different types of genes. *Nᵢ* contigs is the total number of contigs.

MP indicates the likelihood of horizontal gene transfer for a resistance or virulence gene, estimated by its association with MGEs. MP was calculated for each sample as Equation (3):

(3) $$\begin{array}{c} MP_{i}=N_{i}\left(\text{MRGs}/\text{ARGs}/\text{VFGs}-\text{MGEs}\right)/N_{i}\left(\text{MRGs}/\text{ARGs}/\text{VFGs}\right) \end{array}$$

Where *Nᵢ* (MRGs/ARGs/VFGs–MGEs) is the number of genes that were found to be co-located with MGEs. *Nᵢ* (MRGs/ARGs/VFGs) is the total number of genes detected.

MP is an indirect proxy based on co-localization and does not equate to actual transfer frequency. CR, CoR, and MP are expressed as dimensionless ratios. The biological significance of these indices is exemplified by the following: A change of 1 × 10⁻⁵ in CR, CoR, or MP corresponds to an absolute change of one in 100,000​ in the number of gene carriers, microorganisms co-carrying multiple gene types, or MGE-co-located genes, relative to their respective total populations.

### 2.5 Data analysis

Alpha diversity of microbial communities, MRGs, ARGs, and VFGs was evaluated using the Shannon index, richness, and Pielou’s evenness index. Differences between the MS and control groups were compared using Student’s *t*-test and the Wilcoxon rank-sum test. Beta diversity was analyzed via principal coordinate analysis, with PERMANOVA applied to test group differences and to assess the impacts of metals, physicochemical properties, MGEs, and microbial diversity on the composition of communities and genes. The effects of metal and physicochemical properties on the relative abundance and the CR, CoR, and MP of microorganisms and functional genes were examined through regression models and Spearman correlation. Multiple linear regression was applied to data meeting normality and homoscedasticity assumptions; M-estimation was used for data violating these assumptions (e.g., containing outliers); and Bayesian regression was adopted when the preceding methods yielded unreliable results. To prevent model overfitting, weakly informative priors were specified for the Bayesian model: The regression coefficients (β) followed a normal prior distribution, normal (0, 0.5), and the residual standard deviation (σ) followed an exponential prior distribution, exponential(5). Model convergence was evaluated using the Gelman–Rubin statistic, effective sample size (ESS), and Markov Chain Monte Carlo (MCMC) trace plots. Posterior predictive checks were conducted by examining density overlay plots and calculating Bayesian *p*‑values. To control multicollinearity, variables with a variance inflation factor ≥ 5 were excluded. The metal correlation matrix showed a significant positive correlation between Mn and Fe (Fig. S1). Since Mn and Fe were not simultaneously included in any regression model, multicollinearity was effectively controlled. All data processing, statistical analysis, and visualization were conducted using R software (version 4.3.3, R Core Team, R Foundation for Statistical Computing, Vienna, Austria).

## 3 Results

### 3.1 Diversity, composition and distribution of microbial communities, MRGs, ARGs, and VFGs

#### 3.1.1 Diversity of microbial communities, MRGs, ARGs, and VFGs

**Fig. 1:**
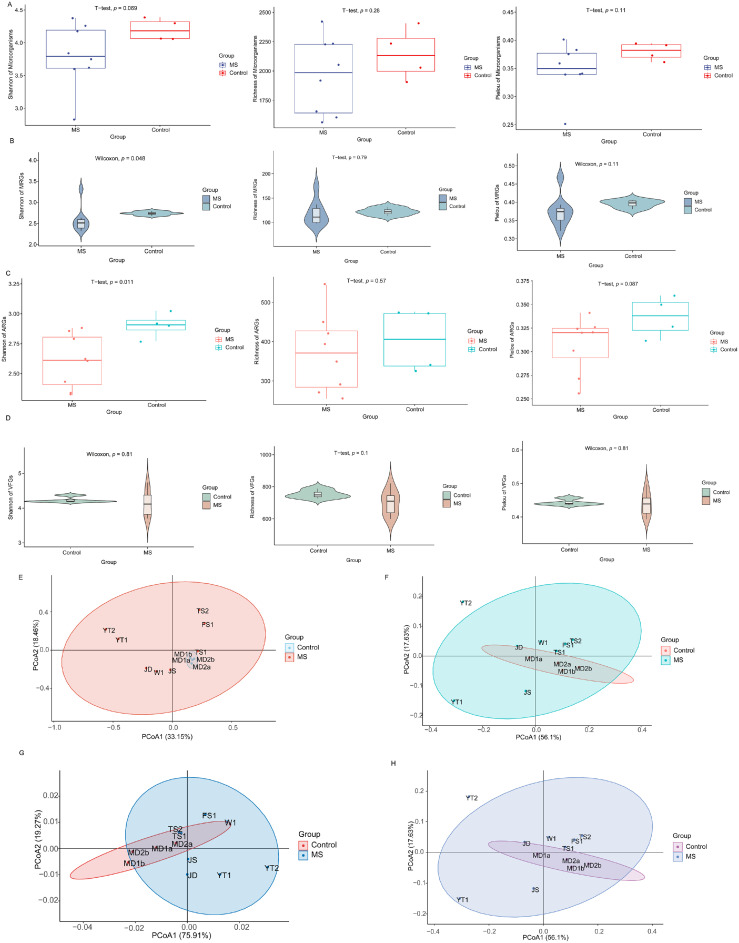
Microbial and functional gene diversity across soil samples. (A) Comparisons of the microbial α-diversity (Shannon index, richness, Pielou) between MS and control groups. (B–D) Comparisons of the α-diversity (Shannon index, richness, Pielou) of MRGs, ARGs, and VFGs, respectively. (E–H) PCoA of microbial communities, MRGs, and VFGs based on Bray-Curtis dissimilarities, and of ARGs based on Horn dissimilarity. MS and control represent the metal-contaminated and uncontaminated control groups, respectively. Statistical significance: * *p* < 0.05; ns, not significant. *Abbreviations*: MRG, metal resistance gene; ARG, antibiotic resistance gene; VFG, virulence factor gene; PCoA, principal coordinate analysis; MS, metal-contaminated soil group.

The alpha diversity analysis demonstrated that the Shannon indices for both MRGs and ARGs were significantly lower in the MS group compared to the control group (*p* < 0.05). No significant differences were observed for the other alpha diversity metrics (*p* > 0.05) (Fig. 1A–D). Principal coordinate analysis combined with PERMANOVA revealed significant differences in the structure of the microbial community (*F* = 2.063, *R*² = 0.171, *p* < 0.05) and the ARG composition (*F* = 11.13, *R*² = 0.527*, p* < 0.05) between the two groups. In contrast, the structures of MRGs (*F* = 1.27, *R*² = 0.11, *p* > 0.05) and VFGs (*F* = 1.64, *R*² = 0.14, *p* > 0.05) showed no significant differences (Fig. 1E–H).

#### 3.1.2 Composition and distribution of the microbial communities

**Fig. 2:**
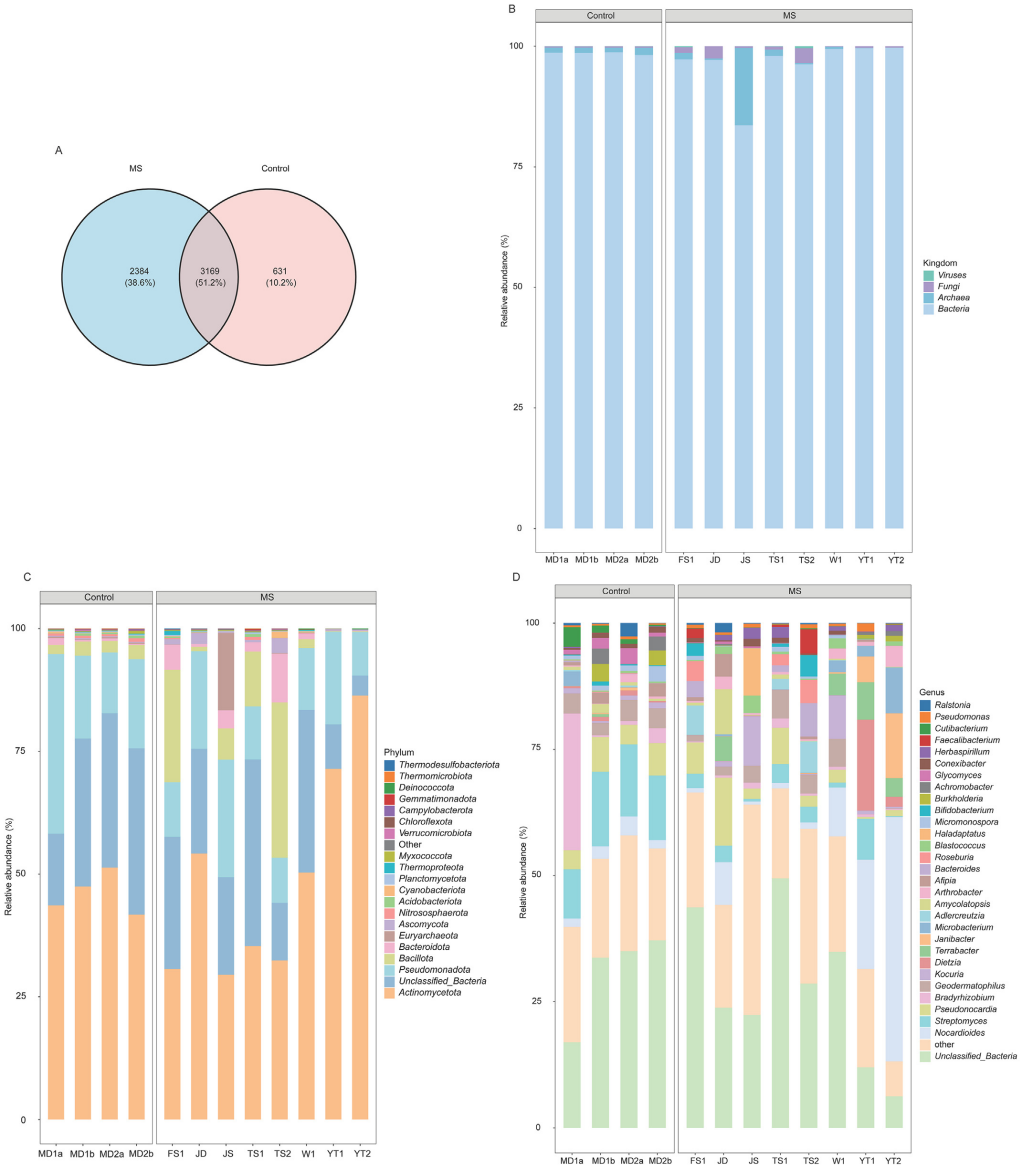

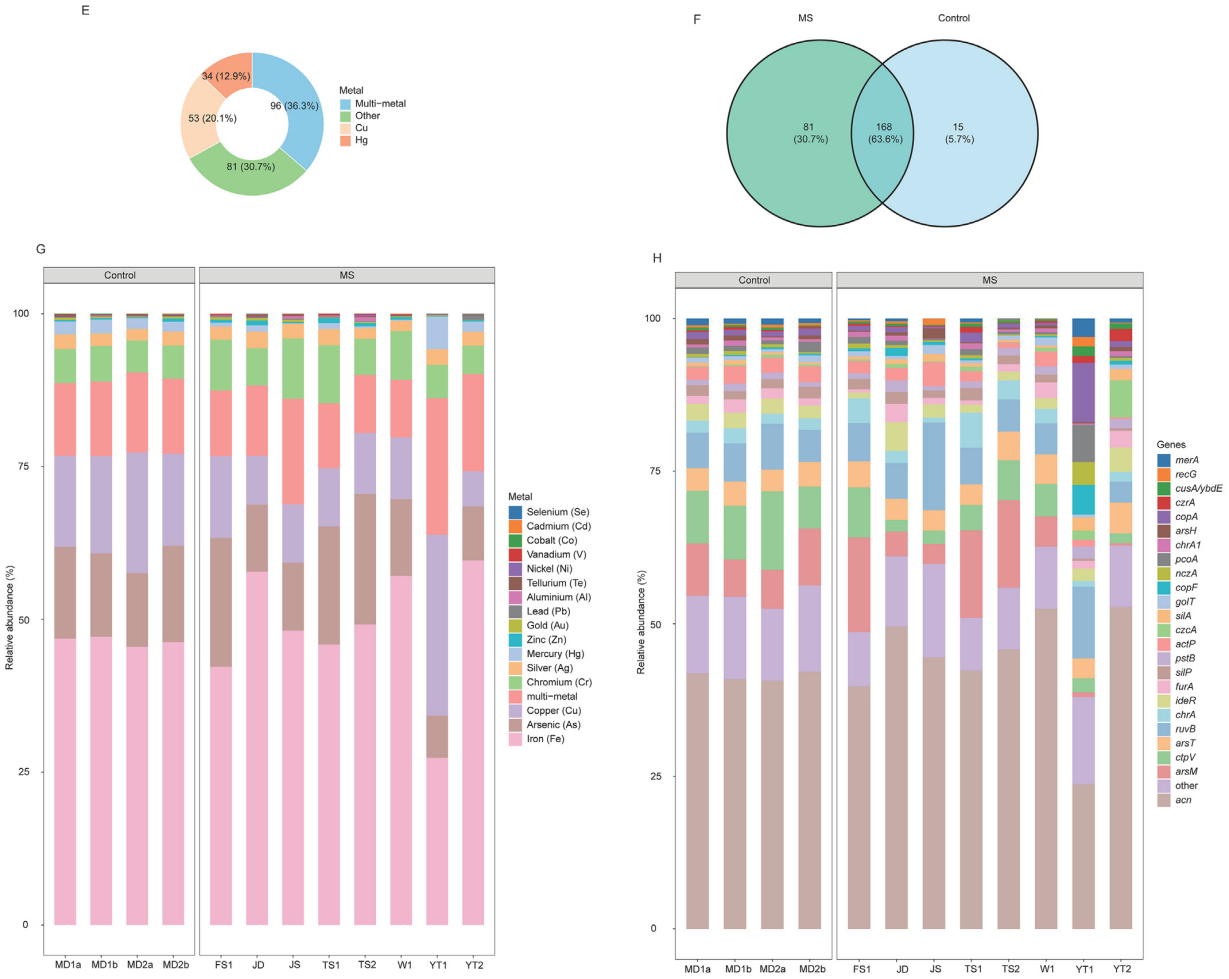
Composition and distribution of the microbial communities and MRGs. (A) Venn diagram showing the numbers of shared and unique microbial species between MS and control groups. The abundances of dominant microorganisms at the (B) kingdom, (C) phylum and (D) genus levels. (E) Relative abundance of MRGs of different resistance types. (F) Number of varieties of MRGs in the MS and control groups. The abundances of dominant MRGs at (G) resistance type and (H) gene levels. MS and control represent the metal-contaminated and uncontaminated control groups, respectively. *Abbreviations*: MS, metal-contaminated soil group; MRG, metal resistance gene.

A total of 6,184 microbial species were detected. Among these, 3,169 species (51.2%) were shared between the two groups, while 2,384 (38.6%) were unique to the MS group and 631 (10.2%) were unique to the control group (Fig. 2A). In the MS group, bacteria, archaea, fungi, and viruses accounted for 96.4%, 2.4%, 1.1%, and 0.1% of the relative abundance, respectively. Notably, the relative abundance of archaea reached 16.0% in one sample (Fig. 2B). At the phylum level, *Actinomycetota* was the most abundant (48.8%), followed by *Unclassified_Bacteria* (20.5%), *Pseudomonadota* (14.4%), and *Bacillota* (9.4%) (Fig. 2C). In the control group, the relative abundances of bacteria, archaea, fungi, and viruses were 98.5%, 1.1%, 0.3%, and 0.1%, respectively (Fig. 2B). The top four dominant phyla were consistent with those in the MS group (Fig. 2C). At the genus level, excluding *Unclassified_Bacteria* (27.6%), *Nocardioides* (11.5%) was the dominant genus in the MS group. In contrast, *Streptomyces* (12.9%) was the most abundant genus in the control group (Fig. 2D).

#### 3.1.3 Composition and distribution of MRGs

A total of 264 MRGs were identified, conferring resistance to 23 metals, including Fe, Cu, Cr, and arsenic (As). Among these, 36.4% were broad-spectrum MRGs, while 20.1% and 12.9% were specific to Cu and mercury (Hg), respectively (Fig. 2E). Shared MRGs between the two groups accounted for 63.6% of the total, while 30.7% were unique to the MS group and 5.7% were unique to the control group (Fig. 2F). In the MS group, the dominant metal resistance types were for Fe (46.4%), As (14.0%), multi-metal (13.4%), and Cu (12.0%). A similar profile was observed in the control group, with relative abundances of 46.4% for Fe, 14.1% for As, 12.4% for multi-metal, and 16.4% for Cu (Fig. 2G). At the gene level, the Fe-resistant gene *acn* was the most abundant in the MS group, accounting for 43.9%, followed by *arsM* (7.2%). In the control group, in addition to *acn* (41.4%) and *arsM* (7.6%), the gene *ctpV* was also dominant, with a relative abundance of 9.3% (Fig. 2H).

#### 3.1.4 Composition and distribution of ARGs

**Fig. 3:**
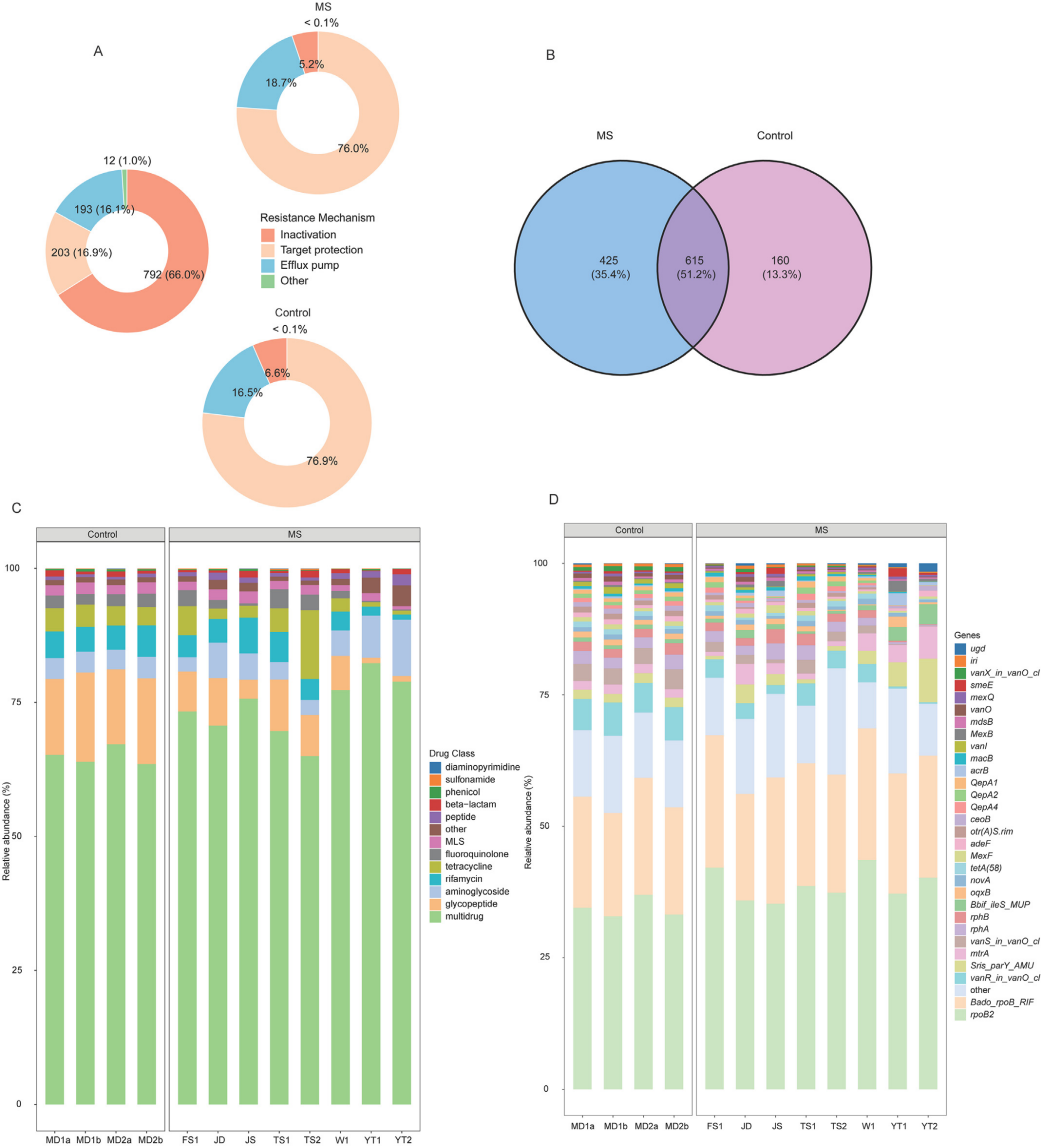

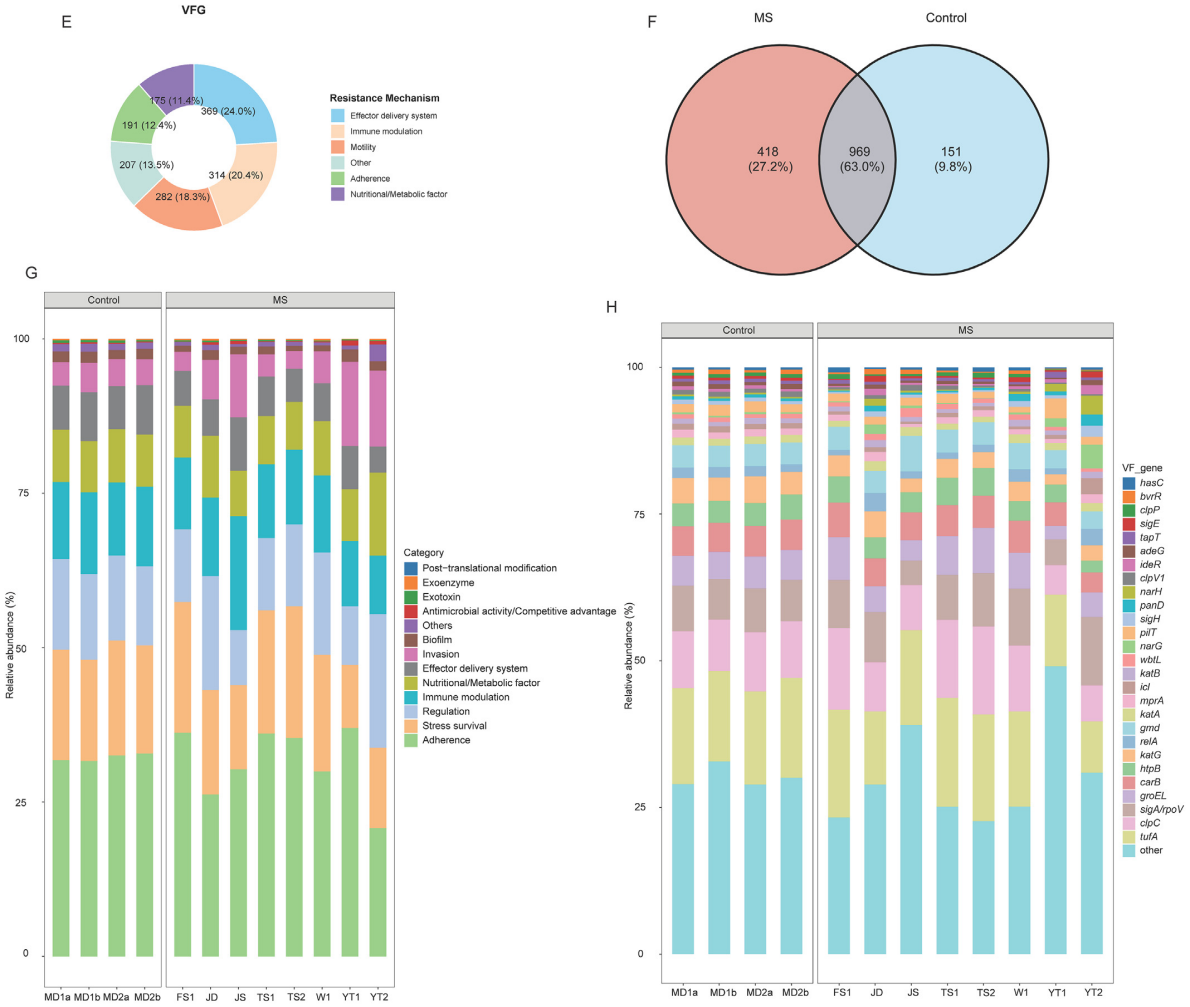
Composition and distribution of ARGs and VFGs. (A) The number of gene types and the relative abundance for ARGs with different resistance mechanisms. (B) The shared and unique ARGs of the MS and control groups. The abundances of dominant ARGs at the (C) resistance type and (D) gene levels. (E) Relative abundance of VFGs of different mechanisms. (F) Number of varieties of VFGs in the MS and control groups. The abundances of dominant VFGs at (G) virulence categories and (H) gene levels. MS and control represent the metal-contaminated and uncontaminated control groups, respectively. *Abbreviations*: ARG, antibiotic resistance gene; VFG, virulence factor gene; MS, metal-contaminated soil group.

A total of 1,200 ARGs were detected. Among these, the primary resistance mechanisms included antibiotic inactivation (66.0%), target protection (16.9%), and efflux pumps (16.1%) (Fig. 3A). Shared ARGs accounted for 51.2% of the total, while 35.4% were unique to the MS group and 13.3% to the control group (Fig. 3B). At the mechanism level, both groups exhibited highly similar profiles, dominated by target protection (MS: 76.0%; control: 76.9%) and efflux pumps (MS: 18.7%; control: 16.5%) (Fig. 3B). Multidrug resistance genes were predominant in the MS group, comprising 74.1% of total ARG abundance, followed by glycopeptide and aminoglycoside resistance genes, each exceeding 5.0%. The top three resistance classes were identical in the control group (Fig. 3C). At the gene level, *rpoB2* (MS: 38.8%; control: 34.4%) and *Bado_rpoB_RIF* (MS: 23.3%; control: 20.8%) were the dominant ARGs in both groups. Additionally, *vanR_in_vanO_cl* was also a major gene in the control group, with an abundance exceeding 5% (Fig. 3D).

#### 3.1.5 Composition and distribution of VFGs

A total of 1,538 VFGs were identified. Among these, the categories of effector delivery system, immune modulation, motility, adherence, and nutritional/metabolic factors each accounted for more than 10% of the total (Fig. 3E). Shared VFGs between the two groups constituted 63.0% of the total, while 27.2% were unique to the MS group and 9.8% were unique to the control group (Fig. 3F). The four dominant virulence mechanisms in the MS group were adherence (31.5%), stress survival (16.9%), regulation (14.0%), and immune modulation (12.4%). An identical profile of dominant mechanisms was observed in the control group, with corresponding proportions of 32.2%, 17.6%, 13.8%, and 12.6% (Fig. 3G). The most abundant VFGs in the MS group were *tufA* (15.1%) and *clpC* (10.1%). The same two genes were also the most abundant in the control group, with relative abundances of 16.2% and 9.6%, respectively (Fig. 3H).

### 3.2 Effects of metal and physicochemical properties on microbial communities, MRGs, ARGs, and VFGs

**Fig. 4:**
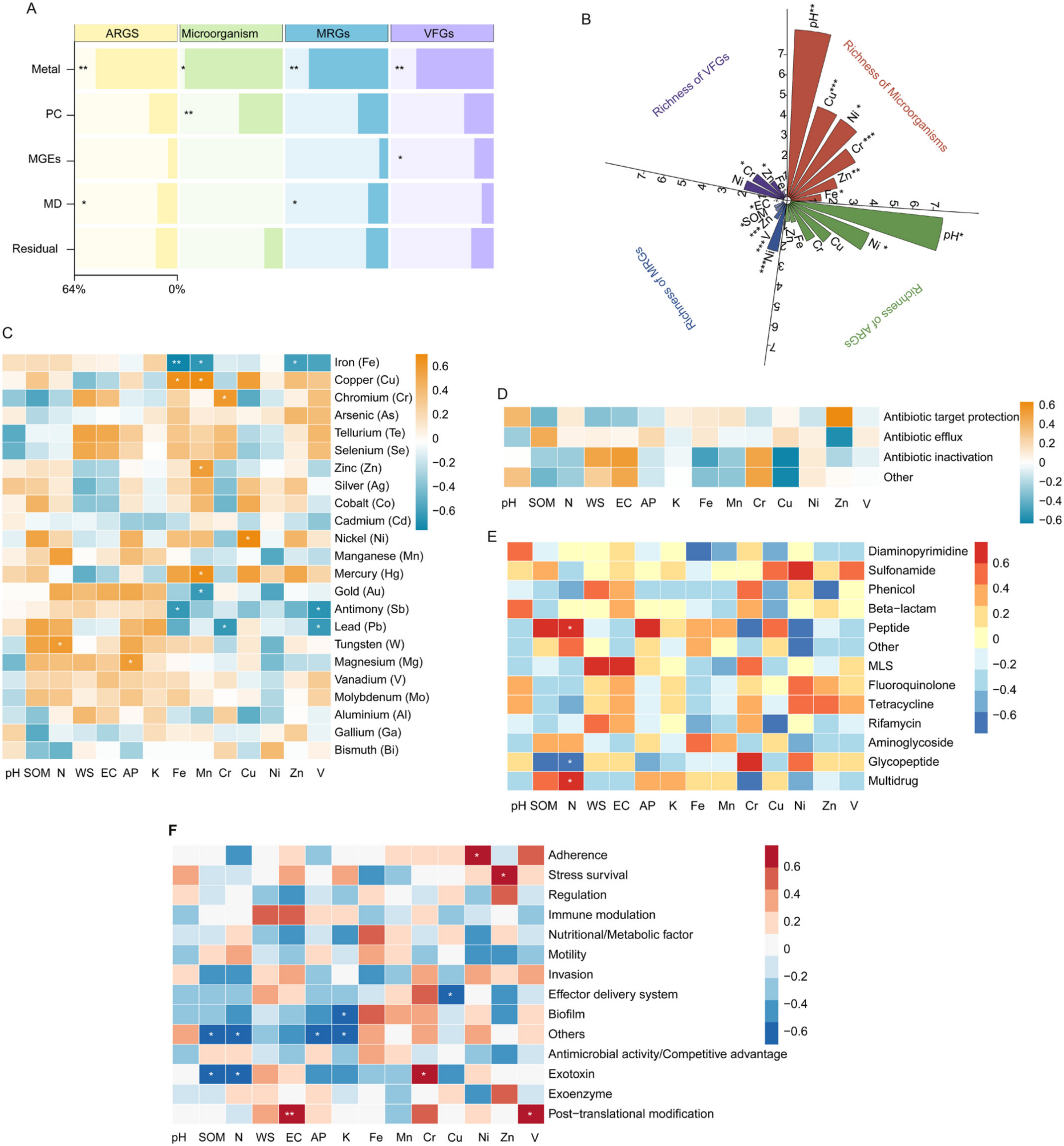
Key drivers of the microbial and functional gene profiles. (A) Contributions of influencing factors to the variation in microbial communities, MRGs, ARGs, and VFGs, PC and MD, respectively. (B) Coefficients of influencing factors on the richness of microorganisms, MRGs, ARGs, and VFGs. (C–F) Heatmaps of Spearman’s correlation coefficients between metals and physicochemical characteristics with the relative abundance of (C) MRGs by type, (D) ARGs by mechanism, (E) ARGs by type, and (F) VFGs by mechanism. *p < 0.05; **p < 0.01; ***p < 0.001. *Abbreviations*: MRG, metal resistance gene; ARG, antibiotic resistance gene; VFG, virulence factor gene; PC, physicochemical characteristics; MD, microbial diversity.

The metal concentrations and physicochemical properties are provided in Supplementary Table S2. PERMANOVA revealed that metal concentration ​​significantly shaped​​ the microbial community, MRGs, ARGs, and VFGs (*p* < 0.05) (Fig. 4A). The M-estimation model (Fig. 4B) revealed the following associations (*p* < 0.05): microbial richness increased by 299.7, 24.6, and 11.6 species per unit increase in pH, Cu, and Cr, respectively, but decreased by 23, 4, and 2 species per unit increase in Ni, Zn, and Fe. Richness of MRGs increased by 0.8 species per mg/kg increase in V, while it decreased by 4.2, 0.5, 0.4, 0.06, and 0.01 species per unit increase in Ni, Zn, SOM, AK, and EC, respectively. For ARGs’ richness, a one-unit increase in pH raised richness by 182.9, and a one mg/kg increase in Ni reduced it by 16.8. Richness of VFGs increased by 2.4 per mg/kg increase in Cr and decreased by 1 per mg/kg increase in Zn.

The relative abundance of Fe-resistant MRGs correlated negatively with Fe, Mn, and Zn concentrations, whereas Fe and Mn levels showed positive correlations with Cu-resistant MRGs (*p* < 0.05). Sb-resistant MRGs were negatively associated with Fe and V concentrations. Mn concentration positively correlated with Zn- and Hg-resistant MRGs but negatively with Aurum (Au)-resistant MRGs. Significant positive correlations included Cr with Cr-resistant MRGs, Cu with Ni-resistant MRGs, N with W-resistant MRGs, and AP with Mg-resistant MRGs, while Pb-resistant MRGs correlated negatively with Cr and V (Fig. 4C). For ARGs, no significant associations were found between soil metal/physicochemical properties and ARGs grouped by resistance mechanism (*p* > 0.05) (Fig. 4D). At the drug class level, the relative abundance of both peptide- and multidrug-resistant ARGs was positively correlated with N concentration, whereas glycopeptide-resistant ARGs showed a negative correlation (*p* < 0.05) (Fig. 4E). For VFGs, EC and V concentration positively correlated with VFGs involved in post-translational modification. Ni and Zn showed positive links to adherence- and stress survival-related VFGs, respectively. Cr correlated positively with exotoxin-related VFGs, while N and SOM correlated negatively. Negative correlations were observed between Cu and effector delivery system VFGs, and between K and biofilm-forming VFGs. Additionally, SOM, N, AP, and K concentrations all negatively correlated with VFGs grouped under other mechanisms (*p* < 0.05) (Fig. 4F).

### 3.3 Effects of metal and physicochemical properties on the CR, CoR, and MP of MRGs, ARGs and VFGs

**Fig. 5:**
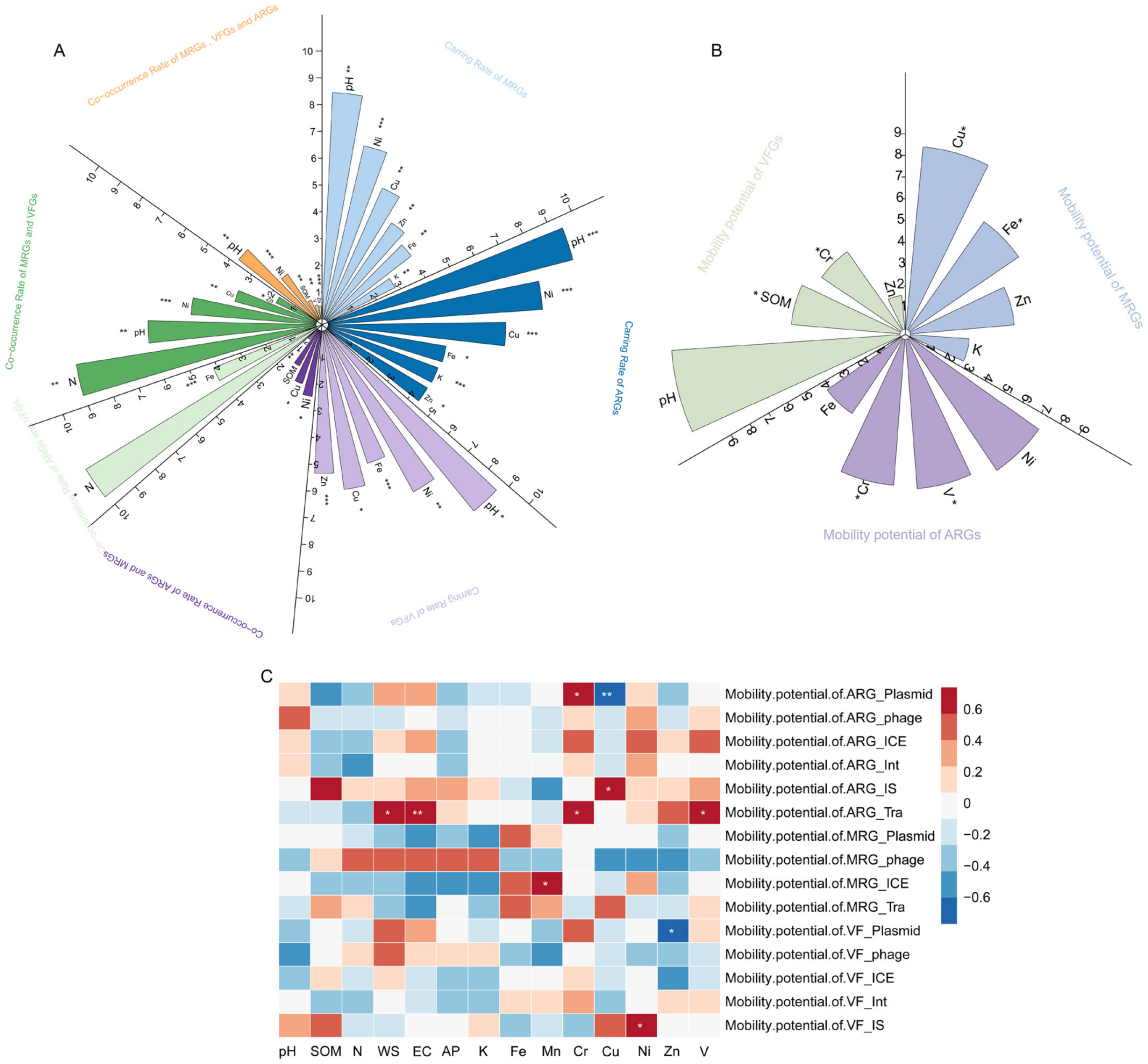
Factors influencing gene carriage, co-occurrence, and mobility potential. (A) Coefficients of influencing factors on the CR and CoR of MRGs, ARGs, and VFGs. (B) Coefficients of influencing factors on MP of MRGs, ARGs, and VFGs. (C) Correlation of metals and physicochemical characteristics with MP of MRGs, ARGs and VFGs mediated by different MGEs. *p < 0.05; **p < 0.01; ***p < 0.001. *Abbreviations*: CR, carrying rate; CoR, co-occurrence rate; MRG, metal resistance gene; ARG, antibiotic resistance gene; VFG, virulence factor gene; MGE, mobile genetic element; MP, mobility potential.

The influences of soil metal contents and physicochemical properties on the CR and CoR of MRGs, ARGs, and VFGs were analyzed using multiple linear regression models (Fig. 5A). Increased pH, Cu, and Fe concentrations significantly elevated the CR of MRGs, ARGs, and VFGs (*p* < 0.05). Per one-unit increase in pH and per 1 mg/kg increase in Cu and Fe resulted in CR increases of 1.4 × 10⁻⁴, 4.3 × 10⁻^3^, and 9.0 × 10⁻⁷ for MRGs; 4.4 × 10⁻⁴, 1.8 × 10⁻⁵, and 1.6 × 10⁻⁶ for ARGs; and 2.0 × 10⁻⁴, 8.7 × 10⁻⁶, and 3.7 × 10⁻⁶ for VFGs, respectively. In contrast, elevated Ni, Zn, and AK concentrations significantly reduced the CR of both MRGs and ARGs. For each 1 mg/kg increase in Ni, Zn, and AK, the CR of MRGs decreased by 1.8 × 10⁻⁵, 1.4 × 10⁻⁶, and 2.3 × 10⁻⁷, respectively, while the CR of ARGs declined by 8.7 × 10⁻⁵, 1.4 × 10⁻⁶, and 1.4 × 10⁻⁶, respectively. Furthermore, each 1 mg/kg increase in Ni and Zn reduced the CR of VFGs by 2.4 × 10⁻⁵ and 4.1 × 10⁻⁶, respectively (*p* < 0.05).

For the CoR, increases of 1 mg/kg in Ni, Cu, and AK reduced the CoR of ARGs and MRGs by 1.5 × 10⁻⁷, 9.7 × 10⁻⁸, and 7.4 × 10⁻⁹, respectively (*p* < 0.05) (Fig. 5A). For the CoR of ARGs and VFGs, increases in N and Fe by 1 g/kg resulted in rises of 9.2 × 10⁻⁴ and 1.1 × 10⁻⁶, respectively (*p* < 0.05) (Fig. 5A). In terms of the CoR of MRGs and VFGs, each unit increase in pH and Cu was associated with increases of 1.2 × 10⁻⁵ and 3.4 × 10⁻⁷, respectively. Conversely, an increase in N by 1 g/kg and increases in Ni and Zn by 1 mg/kg led to reductions of 2.8 × 10⁻⁴, 2.0 × 10⁻⁶, and 5.6 × 10⁻⁸, respectively *(p* < 0.05) (Fig. 5A). Regarding the CoR of MRGs, ARGs, and VFGs collectively, each unit increase in pH, SOM, and V resulted in increases of 6.7 × 10⁻⁷, 1.4 × 10⁻⁸, and 8.3 × 10⁻⁹, respectively. In contrast, increases in Ni and Zn by 1 mg/kg reduced the CoR by 9.4 × 10⁻⁸ and 7.3 × 10⁻⁹, respectively (*p* < 0.05) (Fig. 5A).

Linear regression analysis revealed that soil metal concentrations and physicochemical properties significantly influenced the MP of MRGs (*p* < 0.05) (Fig. 5B). Specifically, for each 1 g/kg increase in Fe concentration, the MP of MRGs was associated with​ an increase of 3.0 × 10⁻⁵, while each 1 mg/kg increase in Cu concentration was correlated with a decrease of 1.1 × 10⁻⁴. Bayesian regression analysis identified V as a significant positive correlate of MP of ARGs, with each 1 mg/kg increase correlating to a 4.7 × 10⁻⁵ rise (95% CI: 2.2 × 10⁻⁵–6.9 × 10⁻⁵). In contrast, each 1 mg/kg increase in Cr was associated with a reduction of 4.3 × 10⁻⁵ in the MP of ARGs (95% CI: −7.4 × 10⁻⁵–9.9 × 10⁻^6^) (Fig. 5B). The Gelman–Rubin statistic for all parameters was 1.00, and the ESS all exceeded 3,700 (Bulk_ESS range: 3,896−5,715; Tail_ESS range: 3,747−5,058). The MCMC trace plots showed good mixing across chains with no evident autocorrelation, indicating that the MCMC sampling had converged adequately and the posterior estimates were reliable (Fig. S2). Density overlay plots demonstrated that the posterior predictive distribution aligned well with the observed data distribution (Fig. S3). The Bayesian *p*‑values of the mean and standard deviation were 0.88 and 0.96, respectively, suggesting that the model provided a good fit in terms of both central tendency and dispersion of the data. For the MP of VFGs, multiple linear regression analysis indicated that each unit increase in SOM and Cr concentration was correlated with increases of 1.7 × 10⁻⁵ and 1.2 × 10⁻⁵, respectively (*p* < 0.05) (Fig. 5B).

Spearman correlation analysis revealed several significant associations (Fig. 5C). For ARGs, the MP of plasmid-mediated types increased with Cr but decreased with Cu, while the MP of IS-mediated types rose with Cu. The MP of transposon-mediated ARGs was positively linked to WS, EC, Cr, and V concentrations. For MRGs, a positive correlation was observed between Fe and the MP of transposon-mediated types. For VFGs, the MP of plasmid-mediated types increased with WS, and the MP of IS-mediated types increased with Ni (all *p* < 0.05).

## 4 Discussion

Heavy metals altered the microbial community structure, at the genus level, the MS group was predominantly characterized by the genus *Nocardioides*, whereas the control group was primarily dominated by *Streptomyces*. The high archaeal abundance in sample JS correlates with its high EC (3966 μs/cm), significantly exceeding other samples (104.7–2,062 μs/cm).^38^ *Nocardioides* is known to be more abundant in nitrogen-rich soils.^39^ The total N content in the MS group (range: 0.041–0.105 g/kg, mean: 0.067 g/kg) was significantly higher than that in the control group (range: 0.03–0.04 g/kg, mean: 0.037 g/kg) (*p* < 0.05) (Fig. S4). The higher relative abundance of *Nocardioides* in the MS group is therefore associated with the higher N content in the soil. In contrast, although *Streptomyces* produces diverse bioactive metabolites, its growth is suppressed by metal stress.^40^ Under the lower metal stress of the control group, *Streptomyces* dominates, fully exploiting its metabolic advantages. The genes conferring resistance to peptide antibiotics and rifamycin (*rpoB2* and *Bado_rpoB_RIF*) were dominant in both the mining-affected and control groups (collectively accounting for over 50% of relative abundance in each group), suggesting their potential role as key candidate genes in heavy metal-driven co-selection for antibiotic resistance.

The metals exerted divergent effects on these genes: for instance, Fe and Cr were positively associated with the proliferation of both MRGs and ARGs, whereas Zn showed an opposite association. Consequently, the observed decrease in diversity within the MS group was likely attributable to the dominance of inhibitory factors, such as Zn. Under high copper stress, the bactericidal effects appear to overwhelm the resistance conferred by *ctpV*, a copper efflux pump gene that was specifically dominant in the control group.^41^ A negative correlation was observed between Fe and Fe-resistant MRGs. While a previous study on park soils reported a positive correlation between Fe and Fe-resistant MRGs,^42^ the Fe levels in our samples (20.5–184.9 g/kg) far exceeded those in the park study (12.5 g/kg). These results suggest that under high metal stress, some microorganisms carrying MRGs may still be unable to withstand the potent bactericidal impact of metals. For ARGs, although certain efflux pump genes were sometimes retained or even enriched due to their non-specificity, intense metal stress likely led to the loss of most non-essential ARGs.^43^ The dominant virulence mechanisms and core VFGs remained highly consistent between the two groups. Previous research has reported that heavy metal contamination significantly affects pathogenic bacteria in low-nutrient environments, while this effect becomes less pronounced under high-nutrient conditions. Therefore, the observed results may be influenced by the nutrient content in the soil.^44^

While higher Fe concentrations were associated with a reduction in microbial richness, they were significantly positively associated with the CR and CoR of MRGs, ARGs, and VFGs. Previous research has confirmed that Fe can promote the proliferation of ARGs.^45^ These findings suggest that even though high concentrations of Fe suppress microbial diversity, they are more likely to trigger active adaptive responses in microorganisms. V concentrations were positively associated with the richness of MRGs, the abundance of adhesion-related VFGs, and the co-occurrence of MRGs, ARGs, and VFGs. This finding supports that vanadium, as a highly toxic multivalent redox-sensitive element, drives co-selection effects by shaping microbial communities and facilitating horizontal gene transfer.^16^^,^^46^^,^^47^ A study reporting a positive correlation between V and microbial abundance documented an average V concentration of 233.57 mg/kg, which is substantially higher than the average concentration in our study (96.04 mg/kg). Furthermore, research has shown that V can exert promoting effects on certain microbial processes at lower concentrations.^48^ This finding is consistent with our observation of predominantly positive effects of V on microbial parameters within this specific concentration range. Cr concentrations were positively associated with the abundance of Cr-resistant MRGs and the richness of VFGs. This aligns with conclusions by Zhang et al.^16^ and Wei et al.,^49^ indicating that Cr can promote the dissemination of the resistome and virulome through co-resistance and cross-resistance mechanisms. Cu concentrations were positively associated with microbial richness, the abundance of Ni-resistant MRGs and exotoxin VFGs, and the CR of MRGs, ARGs, and VFGs. This is closely associated with the ability of Cu to drive the co-selection of MRGs, ARGs, and VFGs through mechanisms such as inducing efflux pump expression.^49^^,^^50^ In contrast, Ni concentrations were significantly negatively associated with microbial richness, the richness of both MRGs and VFGs, and the CR and CoR of various genes. These results are consistent with findings from multiple studies,^51^, ^52^, ^53^, ^54^ indicating that Ni exerts predominant microbial toxicity at elevated concentrations. Similarly, Zn concentrations were negatively associated with microbial richness, MRG richness, the relative abundance of Fe-resistant MRGs, and the CR and CoR of the three gene types. This aligns with reports by Engin et al. regarding the negative correlation between Zn and ARGs, suggesting that high Zn levels exert inhibitory effects by interfering with fundamental enzymatic activities and membrane integrity.^55^^,^^56^

Regarding MP, Fe, V, and Ni significantly enhanced the MP of MRGs, ARGs, or VFGs, while Cr and Cu exhibited bidirectional effects involving both suppression and promotion. Specifically, an increase in Fe concentration was associated with an enhancement in the overall MP of MRGs, particularly correlating with increased transposon-mediated mobilization. This finding aligns closely with the conclusions of Pu et al.,^57^ collectively confirming the role of Fe in driving the dissemination of microbial resistance genes. An increase in V concentration was associated with an increase in the overall MP of ARGs, and was specifically correlated with a higher MP of transposon-mediated ARGs. This result is supported by Suzuki et al.,^46^ who demonstrated that V promotes horizontal gene transfer of ARGs. An increase in Ni concentration was associated with a higher MP of IS-mediated VFGs, a phenomenon explainable by Ni’s key role in virulence regulation in pathogenic microorganisms.^46^ Ni serves as an essential cofactor for various virulence-associated enzymes, which play central roles in host colonization, tissue invasion, and stress resistance of pathogens.^58^ An increase in Cr concentration was inversely associated with the overall MP of ARGs, but positively correlated with the MP of both plasmid- and transposon-mediated ARGs, and was concurrently linked to enhanced transfer potential of VFGs. These results support the perspective proposed by Zhang et al.^16^ that “Cr acts as a co-selective agent driving antibiotic resistance transmission”. Cu concentration was negatively correlated with the MP of plasmid-mediated ARGs, yet showed a significant positive association with the MP of IS-mediated ARGs. This phenomenon is attributed to Cu-induced oxidative stress, which mediates IS mobilization.^59^^,^^60^

This study has several main limitations. Although metagenomic sequencing provided substantial data information, the relatively small sample size (*n* = 12, comprising 8 mining sites and 4 control sites) may limit the statistical power for detecting subtle effects and constrain the application of more complex multivariate models. The absence of temporal data, for instance, precludes tracking the dynamic responses of microbial communities, MRGs, ARGs, and VFGs to metal stress. Future research with sufficient sample sizes​ is needed to provide deeper insights into the ecological mechanisms involved.

## 5 Conclusion

Metals alter the composition and distribution of microbial communities, MRGs, ARGs, and VFGs. A key mechanism underlying this regulation is the modulation of their mobile potential, which either facilitates or restricts horizontal gene transfer.

## CRediT authorship contribution statement

**Qitao Zhang:** Writing – original draft, Visualization, Software, Methodology, Formal analysis, Data curation. **Shixiong Li:** Data curation, Resources, Supervision, Validation. **Xiaobing Wang:** Data curation, Resources, Supervision, Validation. **Yuechen Sun:** Visualization, Data curation, Methodology, Software. **Jingpeng Liu:** Visualization, Methodology, Software. **Jiefei Gao:** Software, Visualization, Data curation. **Chengcheng Deng:** Data curation, Methodology. **Wenlong Zhao:** Methodology, Data curation. **Yixin Ma:** Visualization, Methodology. **Jinrou Quan:** Data curation, Formal analysis. **Qi Yin:** Data curation, Methodology. **Danyan Jian:** Software, Visualization. **Ruihai Zhang:** Data curation, Visualization. **Rui Qi:** Writing – review & editing, Supervision, Software, Resources, Project administration, Methodology, Funding acquisition, Conceptualization.

## Informed consent

Not applicable.

## Organ donation

Not applicable.

## Ethical statement

Not applicable.

## Data availability statement

All metagenomic sequencing data generated in this study have been deposited in the NCBI Sequence Read Archive (SRA) under the BioProject accession number PRJNA1365593. The accession numbers for individual samples are listed in Table S3.

## Animal treatment

Not applicable.

## Generative AI

We confirm that AI-assisted technologies were used solely for language polishing and manuscript refinement. All scientific content, data analysis, and interpretation of results are the original work of the authors.

## Funding

This work was jointly supported by the Jiayuguan City Major Special Science and Technology Program and the Jiuquan City Science and Technology Support Program (24-06 and 2024CA2017).

## Declaration of competing interest

The authors declare that there are no potential conflicts of interest to report regarding the content of this article.

## Appendix Supplementary materials

Image, application 1
