# Supplementary material for: Multi-metal contamination shapes abundance, co-occurrence, and mobility potential of resistance and virulence genes in mining-impacted soils
Source: Infect Med (Beijing). 2026 May 4;5(2):100260. doi: 10.1016/j.imj.2026.100260 (PMC13202555; doi:10.1016/j.imj.2026.100260)
Supplement: Supplementary file 1 [file mmc1.docx]

**Figures**

**
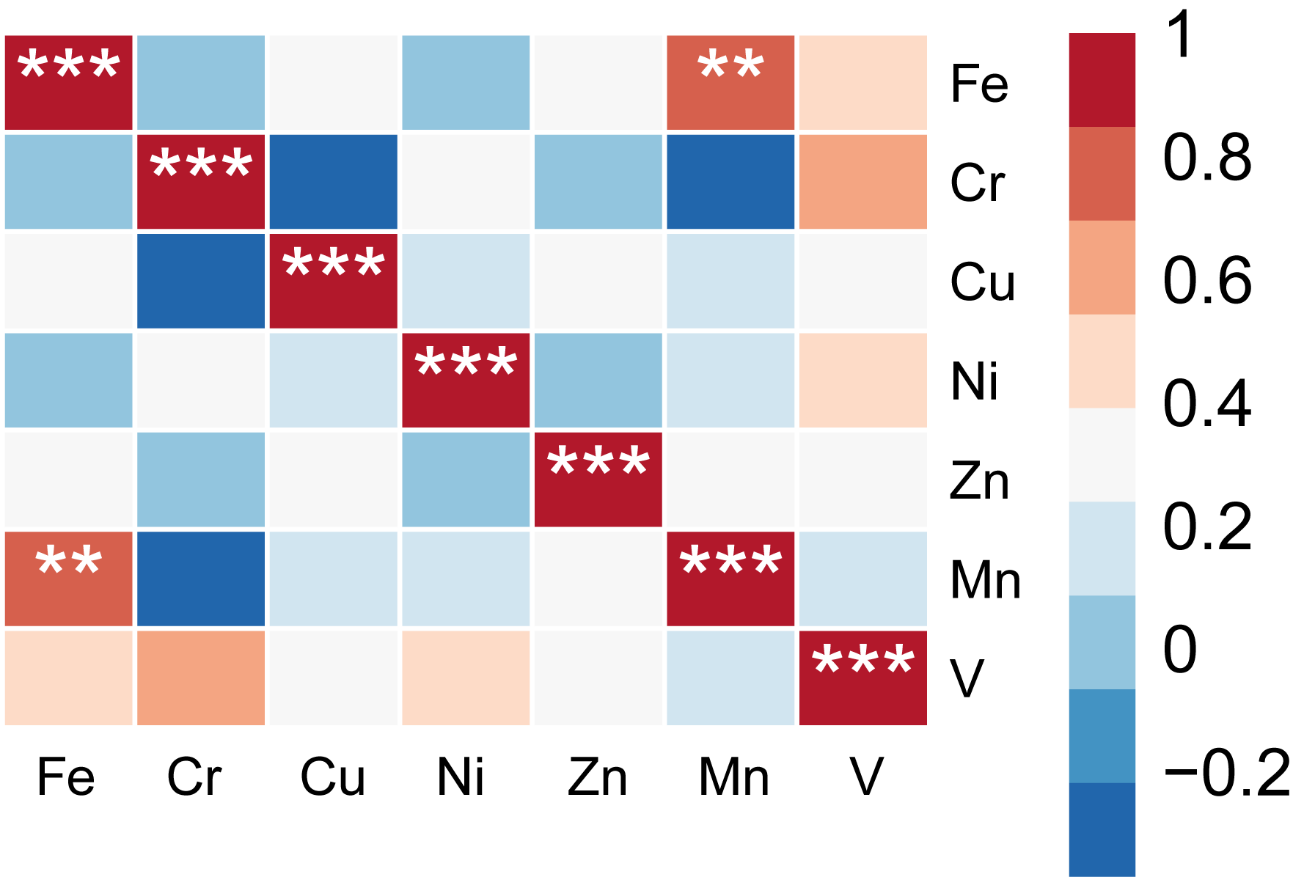
**

**Fig. S1** Heatmaps of Spearman's correlation matrix of metals.

**
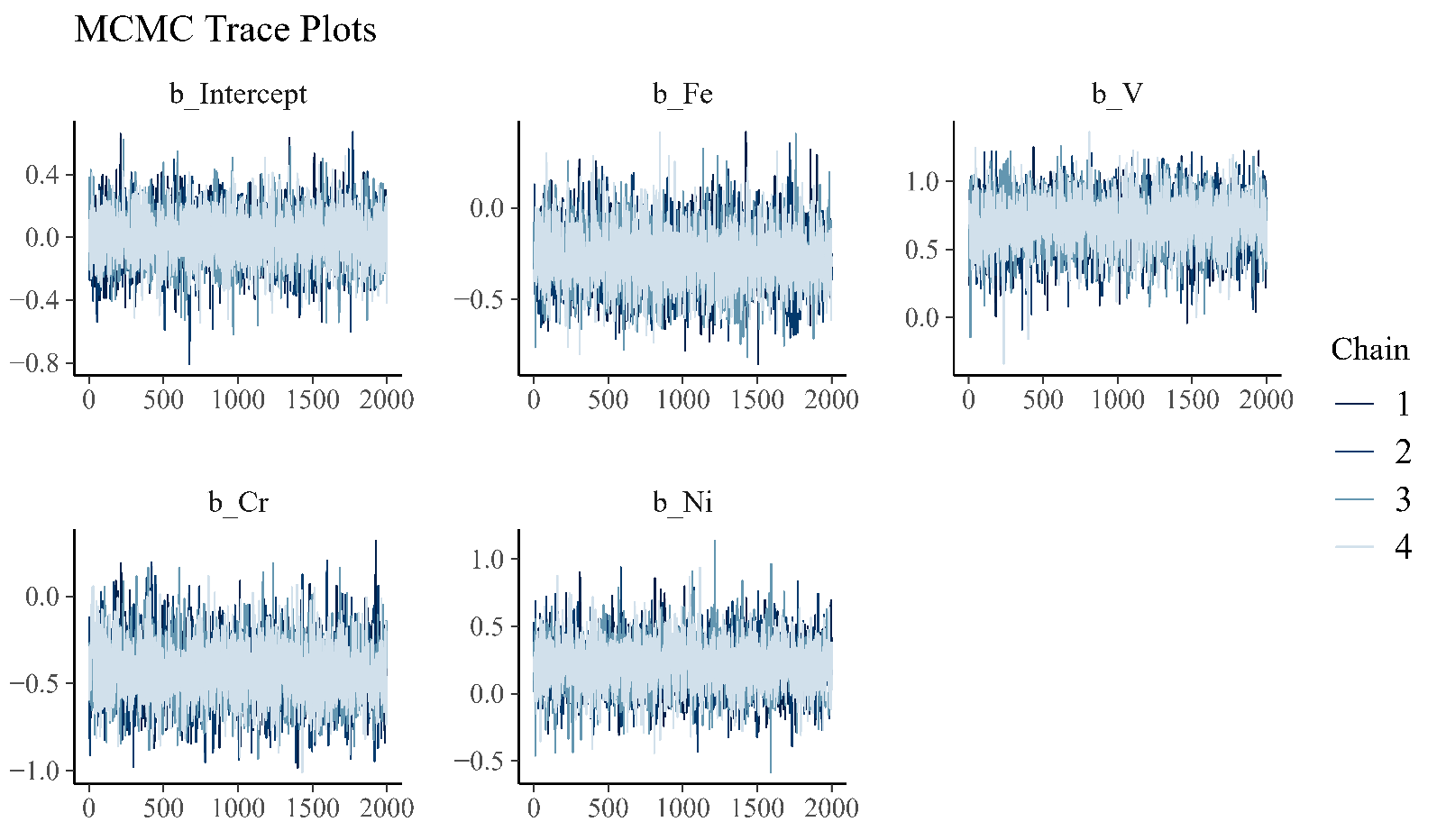
**

**Fig. S2** Trace plots of the Markov chain Monte Carlo (MCMC) for the Bayesian regression model.

**
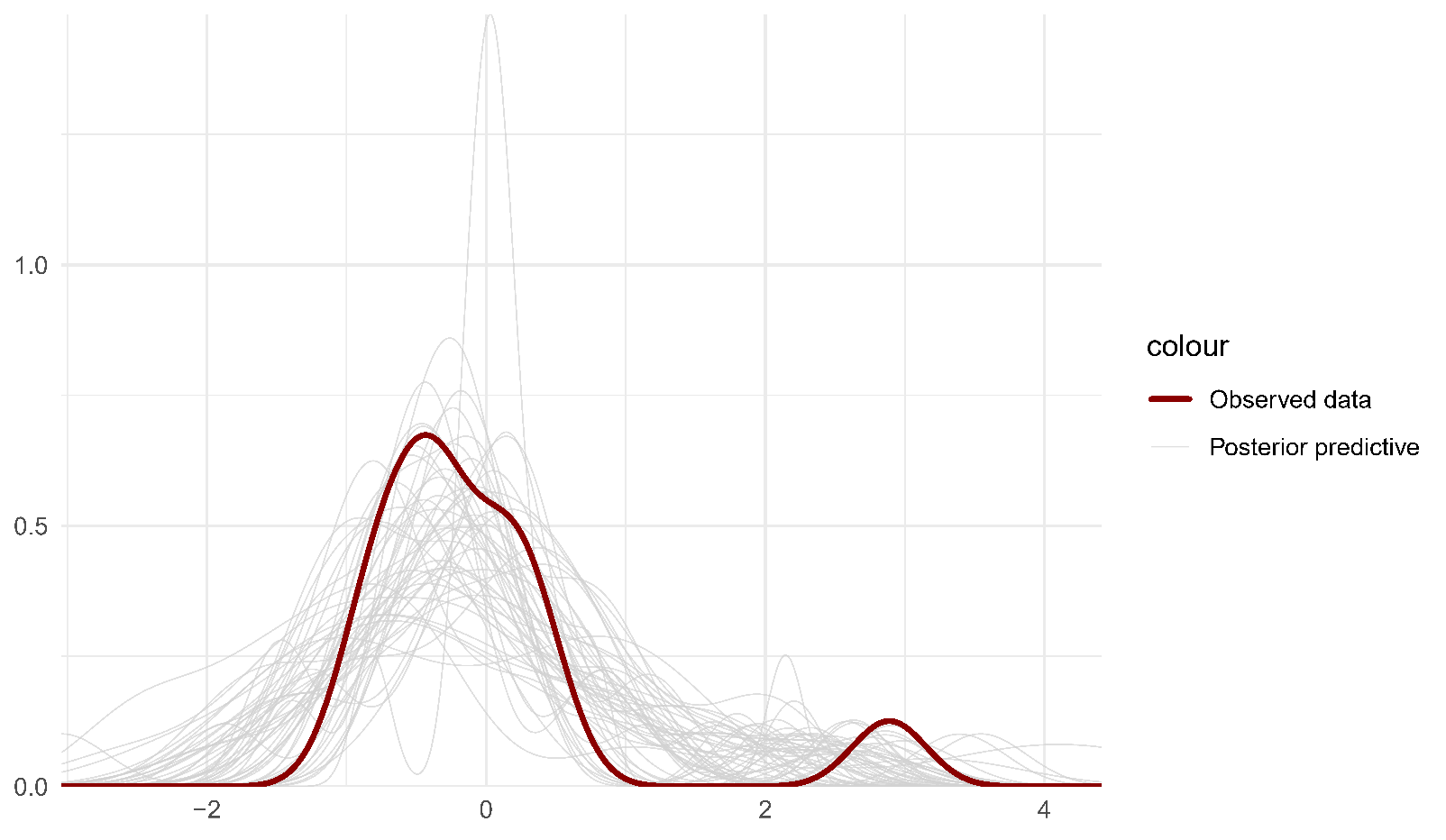
**

**Fig. S3** Density overlay plots for posterior predictive checks of the Bayesian regression model.

**
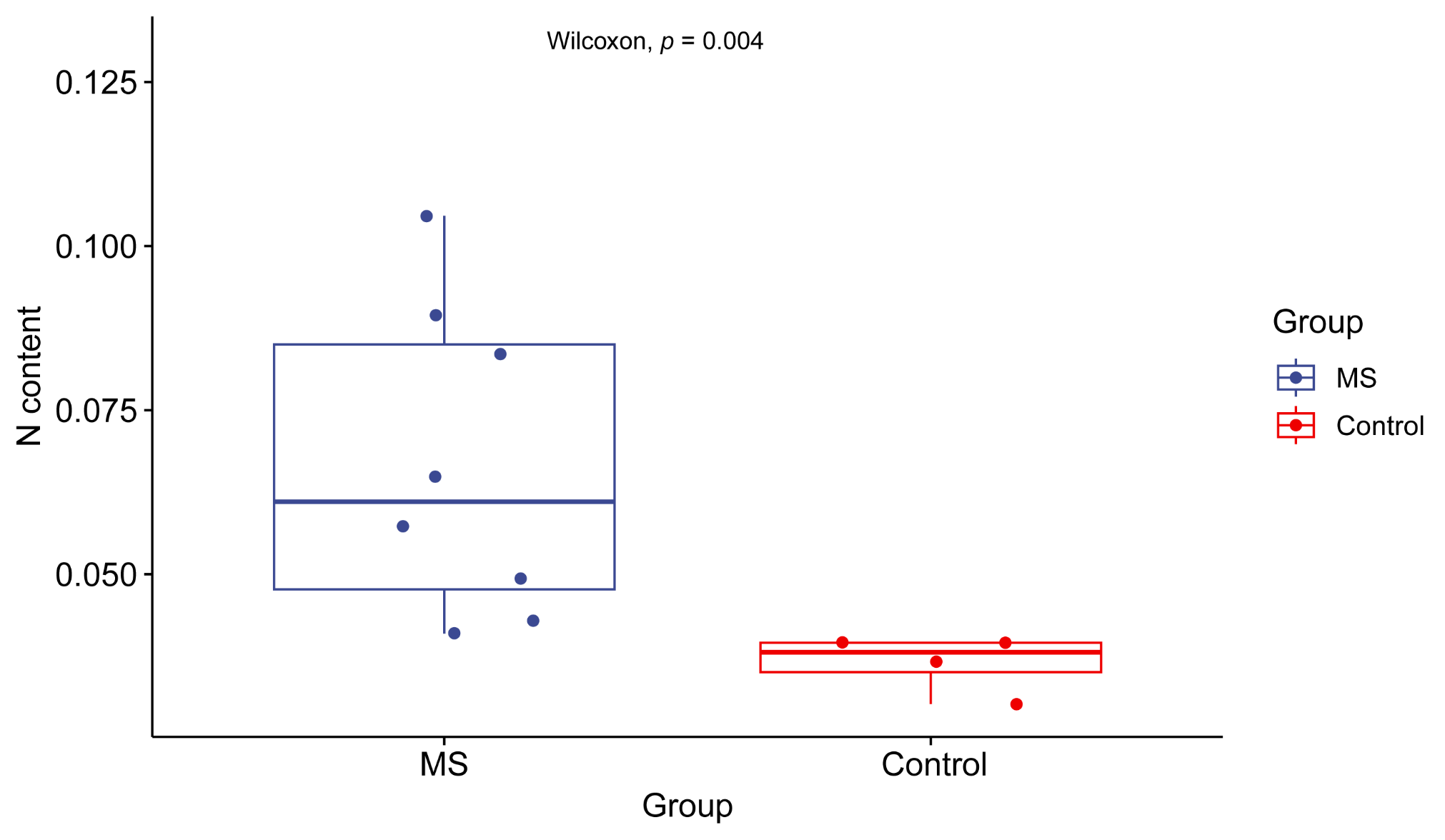
**

**Fig. S4** Comparisons of the soil nitrogen (N) content between MS and Control groups.

**Tables**

**Table S1.** Metal concentrations and physicochemical properties measurement methods.

| soil properties | Measurement Methods |
| --- | --- |
| Fe | ICP-AES (GB15618-2018, China) |
| Cr | ICP-AES (GB15618-2018, China) |
| V | ICP-AES (GB15618-2018, China) |
| Mn | ICP-AES (GB15618-2018, China) |
| Zn | ICP-AES (GB15618-2018, China) |
| Cu | ICP-AES (GB15618-2018, China) |
| Ni | ICP-AES (GB15618-2018, China) |
| pH | electrode method (NYT 1377-2007, China) |
| EC | platinum electrode method (HJ802-2016, China) |
| WS | weight method (NY/T1121.16-2018, China) |
| SOM | potassium dichromate external heating method (LYT1237-1999, China) |
| N | Kjeldahl method (LYT128-2015, China) |
| AP | molybdenum-antimony anti-colorimetric method (LY/T1234-2015, China) |
| AK | flame photometry (LY/T 1234-2015, China) |

WS, water-soluble salts; EC, electrical conductivity; SOM, soil organic matter; AP, available phosphorus; AK, available potassium; N, hydrolytic nitrogen.

**Table S2.** Metal concentrations and physicochemical properties for all samples.

| Group | MS | | | | | | | |  | Control | | | |
| --- | --- | --- | --- | --- | --- | --- | --- | --- | --- | --- | --- | --- | --- |
| Sample | YT1 | YT2 | TS1 | TS2 | FS1 | JS | JD | W1 |  | MD1a | MD2a | MD1a | MD2b |
| WS (g/kg) | 2.64 | 0.58 | 1.24 | 6.80 | 13.22 | 16.01 | 9.54 | 2.95 |  | 7.00 | 7.30 | 7.26 | 9.48 |
| pH | 8.46 | 8.81 | 8.96 | 9.11 | 8.01 | 8.17 | 7.17 | 8.70 |  | 8.68 | 8.80 | 8.77 | 8.56 |
| EC (us/cm) | 474.30 | 104.70 | 173.40 | 2062.00 | 1750.00 | 3966.00 | 1653.00 | 655.10 |  | 1412.00 | 1664.00 | 1622.00 | 1693.00 |
| AP (mg/kg) | 2.70 | 3.41 | 2.40 | 4.33 | 15.52 | 42.18 | 15.80 | 13.48 |  | 1.98 | 1.49 | 2.40 | 2.72 |
| K (mg/kg) | 124.14 | 125.73 | 251.02 | 211.47 | 357.09 | 692.14 | 148.17 | 223.61 |  | 95.08 | 97.93 | 141.78 | 94.90 |
| N (g/kg) | 0.04 | 0.06 | 0.05 | 0.04 | 0.06 | 0.10 | 0.08 | 0.09 |  | 0.04 | 0.04 | 0.03 | 0.04 |
| SOM (g/kg) | 18.66 | 4.37 | 9.56 | 11.83 | 7.43 | 10.37 | 72.96 | 10.04 |  | 1.44 | 1.17 | 1.35 | 1.55 |
| Fe (g/kg) | 184.87 | 51.67 | 20.50 | 38.80 | 42.30 | 33.61 | 37.27 | 45.48 |  | 46.74 | 37.09 | 40.07 | 48.96 |
| Cr (mg/kg) | 44.22 | 42.38 | 14.02 | 49.42 | 49.39 | 47.17 | 43.52 | 40.41 |  | 66.02 | 75.87 | 64.75 | 66.45 |
| Cu (mg/kg) | 47.53 | 42.89 | 26.59 | 47.83 | 43.06 | 20.62 | 50.12 | 29.22 |  | 24.28 | 22.83 | 25.74 | 26.75 |
| Ni (mg/kg) | 34.69 | 25.09 | 34.80 | 40.06 | 30.22 | 22.80 | 24.16 | 23.32 |  | 32.51 | 32.17 | 34.98 | 34.63 |
| Zn (mg/kg) | 52.14 | 61.90 | 58.00 | 112.98 | 77.61 | 46.32 | 58.51 | 192.13 |  | 72.83 | 57.59 | 61.32 | 68.19 |
| Mn (mg/kg) | 2790.16 | 867.35 | 840.43 | 718.42 | 742.25 | 715.19 | 707.03 | 1412.77 |  | 938.09 | 666.33 | 757.16 | 1171.53 |
| V (mg/kg) | 99.28 | 90.88 | 62.27 | 161.74 | 111.84 | 82.41 | 85.45 | 78.85 |  | 93.91 | 87.19 | 93.50 | 105.20 |

Group MS and Control represented metal mine soil and control samples, respectively. WS, water-soluble salts; EC, electrical conductivity; SOM, soil organic matter; AP, available phosphorus; AK, available potassium; N, hydrolytic nitrogen.

**Table S3.​** Accession numbers for the individual metagenomic samples deposited in the NCBI Sequence Read Archive (SRA).

| Sample no. | Sample ID | SRA accession |
| --- | --- | --- |
| 1 | TS1 | SAMN53287365 |
| 2 | TS2 | SAMN53287366 |
| 3 | YT1 | SAMN53287367 |
| 4 | YT2 | SAMN53287368 |
| 5 | JD | SAMN53287369 |
| 6 | JS | SAMN53287370 |
| 7 | FS1 | SAMN53287371 |
| 8 | W1 | SAMN53287372 |
| 9 | MD1a | SAMN53287373 |
| 10 | MD2a | SAMN53287374 |
| 11 | MD1b | SAMN53287375 |
| 12 | MD2b | SAMN53287376 |
